# Specialty Impact on Patient Outcomes: Paving a Way for an Integrated Approach to Spinal Disorders

**DOI:** 10.7759/cureus.45962

**Published:** 2023-09-25

**Authors:** Venkataramana Kuruba, Anjani Mahesh Kumar Cherukuri, Subiksha Arul, Abdulaziz Alzarooni, Sheryl Biju, Taimur Hassan, Riya Gupta, Saya Alasaadi, Jarin Tasnim Sikto, Arnav C Muppuri, Humza F Siddiqui

**Affiliations:** 1 Department of Orthopedic Surgery, All India Institute of Medical Sciences, Vijayawada, IND; 2 Department of Medicine, Guntur Medical College, Guntur, IND; 3 Department of Medicine, JONELTA Foundation School of Medicine, University of Perpetual Help System DALTA, Manila, PHL; 4 Department of Medicine, Royal College of Surgeons Ireland, Dublin, IRL; 5 Department of Medicine, Christian Medical College, Vellore, IND; 6 Department of Medicine, Texas A&M College of Medicine, College Station, USA; 7 Department of Medicine, Shri Atal Bihari Vajpayee Medical College and Research Institute, Bangalore, IND; 8 Department of Medicine, University College of Dublin, Dublin, IRL; 9 Department of Medicine, Jahurul Islam Medical College and Hospital, Bhagalpur, BGD; 10 Department of Medicine, University of Alabama at Birmingham, Birmingham, USA; 11 Department of Internal Medicine, Jinnah Postgraduate Medical Center, Karachi, PAK

**Keywords:** spine fusion, readmission, reoperation, patient outcomes, complications, orthopedic surgery, neurosurgery

## Abstract

Spinal surgical procedures are steadily increasing globally due to broad indications of certain techniques encompassing a wide spectrum of conditions, including degenerative spine disorders, congenital anomalies, spinal metastases, and traumatic spinal fractures. The two specialties, neurosurgery (NS) and orthopedic surgery (OS), both possess the clinical adeptness to perform these procedures. With the advancing focus on comparative effectiveness research, it is vital to compare patient outcomes in spine surgeries performed by orthopedic surgeons and neurosurgeons, given their distinct approaches and training backgrounds to guide hospital programs and physicians to consider surgeon specialty when making informed decisions. Our review of the available literature revealed no significant difference in postoperative outcomes in terms of blood loss, neurological deficit, dural injury, intraoperative complications, and postoperative wound dehiscence in procedures performed by neurosurgeons and orthopedic surgeons. An increase in blood transfusion rates among patients operated by orthopedic surgeons and a longer operative time of procedures performed by neurosurgeons was a consistent finding among several studies. Other findings include a prolonged hospital stay, higher hospital readmission rates, and lower cost of procedures in patients operated on by orthopedic surgeons. A few studies revealed lower sepsis rates unplanned intubation rates and higher incidence of urinary tract infections (UTIs) and pneumonia postoperatively among patient cohorts operated by neurosurgeons. Certain limitations were identified in the studies including the use of large databases with incomplete information related to patient and surgeon demographics. Hence, it is imperative to account for these confounding variables in future studies to alleviate any biases. Nevertheless, it is essential to embrace a multidisciplinary approach integrating the surgical expertise of the two specialties and develop standardized management guidelines and techniques for spinal disorders to mitigate complications and enhance patient outcomes.

## Introduction and background

The exponential increase in the number of procedures performed for degenerative spinal diseases in the elderly population in the past decades has been attributed in large part to expeditiously expanding indications of spine surgical procedures, most commonly spinal fusion techniques [1,2,3]. Degenerative spinal disorders such as disc herniations, spinal stenosis, spondylolisthesis, and myelopathy demand spinal surgery, which encompasses spine arthrodesis and anterior and posterior spine fusion techniques with or without internal fixation [4,5,6]. Lumbar spine fusion, initially developed for spinal tuberculosis, is also becoming widely popular in alleviating chronic lower back pain [6,7]. Anterior lumbar fusion is associated with a soaring postoperative complication rate, reaching as high as 40%, including vascular injury, sepsis, sympathetic dysfunction, and reoperation [8,9]. Anterior cervical discectomy and fusion have been universally accepted as the gold standard procedure for cervical disc degeneration, yet lately, cervical disc arthroplasty is slowly emerging as a preferable alternative due to its numerous advantages. However, complications, including implant migration, insertion failure, surgical site infection, vascular compromise, and cerebrospinal fluid leakage, have been reported [10-13]. The congenital anomalies can lead to complex consequences in adulthood, including defects in vertebral fusion, vertebral segmentation, kyphosis, scoliosis, and kyphoscoliosis [14,15]. Spinal metastases affect the bone tissue and result in spinal instability and may additionally result in epidural compression [16]. The vertebral metastatic tumors likely affecting the cervical spine are treated by percutaneous techniques such as vertebroplasty and palliative procedures comprising decompression and stabilization [17,18].

Traumatic spinal fractures frequently occur as a consequence of road traffic accidents and leisure activities, and the highest proportion of traumatic injuries occur at the cervical level. Traumatic spinal fractures can lead to spinal cord injury (SCI), a mortifying condition resulting in impairment of sensory and motor function, severely diminishing the quality of life [19,20]. An annual increment of up to 15% in surgical procedures for spinal fractures has been recorded persistently [21]. Percutaneous kyphoplasty (PKP) has become a widely acceptable surgical procedure to mitigate pain and halt the advancement of kyphotic deformity among patients of traumatic, osteoporotic, and metastatic compression fractures [22,23]. The two specialties, neurosurgery (NS) and orthopedic surgery (OS), both possess the surgical expertise and adeptness to manage and perform spinal procedures [24].

The steady increase in the age and comorbidities of the patients undergoing spinal procedures has resulted in elevated rates of complications [25,26]. Perioperative complications and hospital readmissions add a substantial burden on the healthcare system; hence, it is imperative to deeply understand the risk factors for these complications [27-29]. The analysis is frequently directed toward complication rates because they not only serve as markers of the quality of care received but also influence resource use, expenditure, and perioperative complications [30, 31]. Previous studies regarding surgical practice in various arenas of surgery have depicted that the same procedure performed by surgeons in distinct specialties attained different outcomes [32,33]. For instance, vascular surgeons encountered a lower mortality rate in abdominal aortic aneurysm procedures compared to general surgeons, while general surgeons were linked to a higher rate of blood transfusions when performing carotid endarterectomy compared to vascular surgeons [33,34]. Lately, healthcare reforms have placed a significant emphasis on comparative effectiveness research to delineate the most effective therapies under various conditions [35].

Due to the well-established differences in training between neurosurgeons and orthopedic surgeons with respect to duration, case volumes, and fellowship pathways, there is a perceived polarity in operative performance and patient outcomes in spinal procedures [36]. Studies show that during their residency training, NS residents gain three times more exposure to spinal surgical procedures in comparison to OS residents. Generally, spine surgery constitutes approximately 37% of cases of NS training compared to only 16% of OS training. Furthermore, NS residents expressed a greater level of confidence in their potential to operate on the spine after their training in contrast to OS residents [37-40]. Fundamental differences continue to proliferate between the two specialties regarding the management of spinal conditions. Neurosurgeons prefer to utilize variable imaging modalities more frequently to diagnose spinal fractures and advocate the use of bracing twice as often compared to orthopedic surgeons [41,42]. In recent times, the increased accessibility to data regarding patient and hospital performance and enhanced patient autonomy in the physician selection process necessitates that surgeons profoundly understand the patient’s expectations and manage them accordingly [43].

A certain level of disparity exists in the available literature concerning the impact of NS and OS specialties on the outcomes of spinal surgical procedures. Some studies have invariably maintained that past surgical training has no consequence on perioperative complications, while other studies showed notable differences in complication rates and outcomes. Unfortunately, biased studies establishing the superiority of one specialty over the other have been disseminated undermining the significance of the collaboration of NS and OS [24]. A collaborative approach in training spine surgeons under the mentorship of both neurosurgeons and orthopedic surgeons is critical and will equip the trainees with multifaceted skills [44]. The review aims to compare the patient outcomes of spinal surgical procedures between NS and OS to guide hospital programs and physicians to consider surgeon specialty when making decisions and formalize management guidelines integrating the two specialties to mitigate complications and achieve favorable outcomes. 

## Review

Spinal surgery is considered a critical territory within both orthopedic and neurosurgical practices. Spinal surgeries are usually performed to diminish pain, reconstruct specific areas for better function, and correct deformities, to allow a better quality of life for patients affected by spinal disorders that impact the vertebral column and associated structures [24,25,26]. Studies outlining differences between the two specialties in the perioperative outcomes among spinal surgery patients have been presented in Table 1.

**Table 1:** Perioperative outcomes among spinal surgery patients between OS and NS. NS, neurosurgery; OS, orthopedic surgery; ACS-NSQIP, American College of Surgeons–National Surgical Quality Improvement Program; ALIF/LLIF, anterior lumbar interbody fusion/lateral lumbar interbody fusion

Author and journal	Study database	Procedure type	Study period	No. of NS patients	No. of OS patients	Primary outcomes	Findings
Alomari et al., 2021 World Neurosurgery [46]	ACS-NSQIP	Single-level and multi-level anterior/lateral lumbar interbody fusion	2015-2018	4,801	4,269	30-day outcome	Patients undergoing ALIF/LLIF performed by orthopedic surgeons had a higher rate of perioperative blood transfusions, return to the operating room within the same admission, were discharged to a destination other than home, and often high higher readmission rates.
Alomari et al., 2022 Neurosurgery [51]	ACS-NSQIP	Single level and multilevel Elective Anterior Cervical Diskectomy and Fusion	2016-2018	15,658	5,553	30-day outcome	Longer operative time, shorter hospital stays, and lower rates of return to operating room, nonhome discharge, discharge after postoperative day 1, perioperative blood transfusion and sepsis in NS patients. In the single-level group, lower readmission and unplanned intubation rates in NS patients.
Baek et al., 2019 World Neurosurgery [47]	Humana Administrative Claims	One- to two-level posterior lumbar fusion	2007-2015	5,523	4,986	90-day outcome	No statistically significant difference
Bronheim et al., 2018 World Neurosurgery [45]	ACS- NSQIP	Anterior lumbar fusion	2010-2014	1,629	1,553	30-day outcome	Although no significant differences were found between the two specialties, surgeries performed by neurosurgeons had a higher rate of reoperation and urinary tract infection.
Chun et al., 2018 Clinical Spine Surgery [58]	Centers for Medicare and Medicaid service and ProPublica Surgeon Scorecard	Elective spinal arthrodesis	2011-2013	2,110	2,110	30-day outcome	No statistically significant difference.
Esfahani et al., 2018 World Neurosurgery [48]	ACS-NSQIP	Single-level lumbar discectomy	2005-2014	3,733	8,926	30-day outcome	Neurosurgeons had a slightly longer mean operative time. Orthopedic surgeons had slightly higher blood transfusion rates, which were insignificant after the Bonferroni adjustment.
Hu et al., 2019 BMC Surgery [59]	ACS-NSQIP	Single-level percutaneous kyphoplasty	2012-2014	1,229	1,019	30-day outcome	Higher rate of postoperative blood transfusion in OS patients
Kim et al., 2014 Spine [49]	ACS-NSQIP	Single-level lumbar fusion	2006-2011	1,264	1,264	30-day outcome	No statistically significant difference
Malik et al., 2020 Clinical Neurology and Neurosurgery [61]	Humana Administrative Claims	Fusions, laminectomies, or osteotomy/corpectomy for spinal metastases	2007-2017	683	204	90-day outcome	No statistically significant difference
Mabud et al., 2017 Clinical Spine Surgery [55]	Thomson Reuters MarketScan Commercial Claims	Lumbar laminectomy, lumbar fusion, lumbar laminectomy with fusion, and anterior cervical discectomy and fusion	2006-2010	99,615	98,697	30-day outcome	Differences were minimal between the two specialties. However, patients operated on by orthopedic surgeons were more likely to receive perioperative transfusions and an increased length of stay.
McCutcheon et al., 2015 Spine [57]	ACS-NSQIP	Spinal fusion	2005-2011	5,247	4,350	30-day outcome	No statistically significant difference
McDonald et al., 2023 Spine Journal [60]	PearlDriver Mariner	Spinal deformity procedures	2010-2019	4,063	8,866	5-year outcome	Orthopedic surgeons performed the majority of the procedures and had lower average costs. Neurological surgeons operated on a greater number of older patients with more comorbidities.
Minhas et al., 2014 Spine [50]	ACS-NSQIP	Single-level anterior cervical discectomy and fusion	2006-2012	1,557	387	30-day outcome	No statistically significant difference
Myers et al., 2021 British Journal of Neurosurgery [62]	Trauma Audit and Research Network, single institution	Spinal fracture management	2012-2016	266	199	Length of stay, complications till discharge, mortality	Overall complication rate was higher in OS patients.
Gupta et al., 2022 Global Spine Journal [54]	ACS-NSQIP	Single-level cervical disc arthroplasty	2015-2020	2,263	916	30-day outcome	No statistically significant difference
Prabhakar et al., 2020 Journal of Orthopedics [52]	ACS-NSQIP	Anterior cervical discectomy and spinal fusion	2011-2014	13,356	4,611	30-day outcome	No statistically significant difference in the 30-day outcome. Slightly longer operative time in NS patients.
Seicean at al., 2014 Spine [56]	ACS-NSQIP	Elective spinal fusion and laminectomy	2006-2012	33,235	17,126	30-day outcome	Increased rate of perioperative blood transfusion and slightly longer hospital stay in OS patients.
Snyder et al., 2018 Spine [53]	ACS-NSQIP and single institution data	Posterior cervical decompression and fusion	2006-2016	9,389	2,548	30-day outcome	Orthopedic patients have higher rates of perioperative blood transfusion and in-hospital complications.

Lumbar discectomy and fusion

Bronheim et al. conducted a study to assess the discrepancies in outcomes between patients undergoing anterior lumbar fusion (ALF) performed by orthopedic spine surgeons and neurosurgeons. The study encompassed a cohort of 3,182 patients from the National Surgical Quality Improvement Program (NSQIP), divided into 1,629 (51.2%) patients in the NS group and 1,553 (48.8%) patients in the orthopedic group. Neurosurgeons used intravertebral devices (74.2% vs. 65.4%, *P* < 0.001) and bone grafts (54.1% vs. 44.9%, *P* < 0.001) more frequently during intraoperative ALF procedures. The results showed that there was no statistically significant difference between NS and OS in 30-day postoperative complications. These complications encompass various aspects, including the length of stay for more than five days, discharge destination, wound complications, cardiopulmonary deteriorations, renal abnormalities and urinary tract infections (UTIs), intraoperative or postoperative blood transfusion, sepsis, reoperation rates, unplanned readmission, and mortality [45].

A study performed by Alomari et al., focusing on the effect of surgeon specialty on lumbar fusion surgery using the NSQIP database, analyzed 9,070 patients. Among them, 4,801 (52.9%) were in the NS group and 4,269 (47.1%) were in the OS group. Patients undergoing anterior lumbar interbody fusion (ALIF) and lateral lumbar interbody fusion (LLIF) performed by orthopedic surgeons had greater rate of return to the operating room within the same hospitalization, had higher readmission rates and greater rate of perioperative blood transfusions, and were transferred to another facility rather than home more often, compared to NS patient cohort. However, lesser operative duration was measured among surgeries performed by orthopedic surgeons. There was no statistically significant difference in multilevel fusion procedures [46].

The study performed by Baek et al. included posterior lumbar fusion (PLF) surgeries performed by both orthopedic and neurological surgeons. Based on the 2008-2015Q2 Humana Commercial Database, the study identified 10,509 patients who specifically underwent 1- to 2-level PLFs. Complications and costs were measured for both specialties and compared using sophisticated statistical analyses. Between the two patient groups, there were no statistically significant differences in 90-day complication rates; however, slightly higher wound complications and dural tears were found in the orthopedic group. No statistically significant differences were noted in the costs between the two groups, except that surgeon reimbursement was lower for OS versus NS. The authors concluded that the provider’s specialty does not markedly influence the 90-day surgical outcome and costs after elective PLFs but encouraged a dual training pathway for spine surgeon specialty [47].

Esfahani et al. assessed the influence of surgical specialty on 30-day postoperative complication rates for single-level lumbar discectomies among propensity-matched cohorts. Patients who underwent laminotomy (hemilaminectomy) for decompression of nerve roots, including partial facetectomy, foraminotomy, or excision of herniated intervertebral disc procedures, were included to confine simple, single-level lumbar discectomy cases. Patients receiving blood transfusions, 0.3% (*N *= 11) of orthopedic patients and 0.1% (*N *= 3) of NS patients, were the only statistically significant (*P* = 0.032) postoperative outcome difference between the two cohorts. Nevertheless, after the Bonferroni adjustment was applied, the correlation between surgeon specialty and blood transfusion was no longer significant. This could be due to a difference in emphasis on the maintenance of hematocrit levels in the postoperative period during training of the different specialties, or it could depend upon the willingness of the surgeon to operate on anemic patients. It may be due to chance alone, as the incidence of blood transfusion in this study is low (0.18%). The operative time was slightly longer in the case of neurosurgeons (83.7 minutes) compared to orthopedic surgeons (72.5 minutes; *P* < 0.001). The reason for this is unclear and could be related to differences in the use of minimally invasive techniques or the presence of residents in the operative room, both of which are not studied in the NSQIP database. Mortality was seen in two NS patients, which is a statistically insignificant difference from the OS patients in which no mortality was observed [48]. Kim et al. assessed 30-day complication rates among 1,263 matched pairs of patients undergoing single-level lumbar fusions operated by neurosurgeons and orthopedic surgeons. Posterior or posterolateral and posterior interbody techniques were the most frequently performed procedures in both the unadjusted and the propensity-matched populations. The multivariate analysis showed that the orthopedic surgery cohort (OC) had no statistically significant differences in odds ratios (OR) for the development of any complication compared with the neurosurgery cohort (NC) [49].

Cervical discectomy and fusion

Minhas et al. were able to acumen the influence of surgeon specialty on 30-day postoperative complication rates following a single-level anterior cervical discectomy and fusion (ACDF) extracting data from the NSQIP database. Propensity scored matching (PSM) was performed to reduce the biases. Out of 1,944 total patients, 19.9% were operated on by orthopedic surgeons and 81.1% by neurosurgeons. Analysis exhibited that before the PSM was conducted, results showed that patients who had undergone surgery by neurosurgeons had a higher rate of complications compared to those treated by orthopedic surgeons. However, after the PSM, no statistically significant difference in postoperative complications was found between the two specialties [50].

Alomari et al. explored the impact of spine surgeon specialty on early perioperative outcome measures of elective ACDF. The study included 11,209 single-level ACDF cases, out of which there were 8,373 (74.7%) patients in the NC and 2,836 (25.3%) patients in the OC. 10,002 multilevel ACDF cases were included, out of which there were 7,285 (72.8%) patients in the NC and 2,717 (27.2%) patients in the OC. In both single-level and multilevel cohorts, patients operated on by neurosurgeons were more likely to have a longer operative time, shorter total hospital stay, lower rate of return to the operating room within the same admission, discharged to home more frequently, lower discharge after postoperative day 1, lower perioperative blood transfusion rate, and lower sepsis rates. In the single-level ACDF cohort, patients operated on by neurosurgeons were more likely to have lower readmission rates and unplanned intubation rates compared to OS [51]. Prabhakar et al. presented the findings of their study evaluating the differences in outcomes between patients undergoing ACDF performed by orthopedic spine surgeons and neurosurgeons yielding similar results. The study reviewed 17,967 patients from the NSQIP. These were then divided into two groups: 4,611 (25.6%) were orthopedic patients and 13,356 (74.3%) were neurosurgical patients. The results indicate a lack of significant difference between orthopedics and neurosurgeons in 30-day postoperative complications. These complications include length of hospital stay, ventilator dependency for more than 48 hours, and rehospitalizations. However, operative times were slightly longer in the NS patients [52].

A comprehensive analysis was conducted to compare the rates of in-hospital complications for patients undergoing posterior cervical decompression and fusion (PCDF) performed by orthopedic spine surgeons and neurosurgeons. The study examined the data of 12,221 patients: 1,221 patients from a single institution and 11,116 patients from the NSQIP database. In both cohorts, a larger number of patients underwent PCDF performed by neurosurgeons than orthopedic surgeons (single institution: 55.94% vs. 44.06%; NSQIP: 78.32% vs. 21.68%). Comparison of in-hospital complications in the single institution cohort revealed that patients who underwent PCDF performed by orthopedic surgeons had a higher prevalence of bleeding necessitating transfusions (14.5% vs. 9.08%, *P* = 0.003) as well as pulmonary embolism (0.74% vs. 0.00%, *P* = 0.04). Conversely, patients in the NS group had a higher occurrence of airway complications (1.17% vs. 0.00%, *P* = 0.01) and pneumonia (4.25% vs. 0.74%, *P* = 0.0002). Comparison within the NSQIP database also showed that patients in the orthopedic cohort exhibited an increased risk of bleeding necessitating transfusions (11.16% vs. 6.18%, *P* < 0.0001) as well as septic shock (0.71% vs. 0.32%, *P* = 0.009). On the other hand, patients treated by neurosurgeons experienced a higher occurrence of deep vein thrombosis (0.76% vs. 0.37%, *P* = 0.04). Analysis of the NSQIP database revealed that patients in the orthopedic cohort were 1.66 times more likely to have an in-hospital complication compared with patients in the NS group [53].

Gupta et al. also examined 30-day complications for patients who underwent single-level cervical disc arthroplasty (CDA) performed by neurosurgeons as opposed to orthopedic surgeons. The study examined 3,179 single-level CDA patients from 2015 to 2020, with 28.8% handled by orthopedic surgeons and 71.2% by neurosurgeons. After balancing variables and adjusting for confounders, no significant difference was found between the orthopedic and neurosurgical specialties [54].

Cervical, thoracic, and lumbar fusion

Mabud et al. compared retrospectively the outcomes of lumbar laminectomy, lumbar fusion, lumbar laminectomy with fusion, and ACDF among 197,682 patients. They found that postoperative complications were comparable between neurosurgeons and orthopedic surgeons, but marginally higher for NS-treated patients undergoing lumbar fusion (OR 1.14; 95% confidence interval [CI] 1.09-1.12) and ACDF (OR 1.09; 95% CI 1.04-1.15). No statistically significant differences were found between the type of surgeon and 30-day readmission rates. However, revision rates for laminectomy with fusion (OR 1.14; 95% CI 1.08-1.22) and ACDF (OR 1.20; 95% CI 1.14-1.28) were slightly higher in the neurosurgical group, possibly due to more severe comorbidities. Data were extracted from the Market Scan database, which depends on accurate coding and may be susceptible to biases as it lacks clinical details [55].

Seicean et al. accomplished a retrospective analysis of data from NSQIP and compared the outcomes of elective spine fusion and laminectomy performed by neurosurgeons and orthopedic surgeons among 50,361 patients operated between 2006 and 2012. They revealed that the patients undergoing OS were more than twice as likely to have prolonged hospital stays and higher perioperative transfusions, along with slightly increased complications and continued care needs, compared to NS patients. Additionally, the study observed that patients treated by orthopedic surgeons stayed in the hospital for a median of three days, while those treated by neurosurgeons stayed for two days. However, there were minimal variations observed in other postoperative outcomes such as 30-day readmission and 30-day mortality between the two surgical specialties [56].

McCutcheon et al. attempted to compare differences in return to the operating room, mortality, and 30-day perioperative outcomes for patients undergoing spinal fusion surgery between two specialties. The surgical procedures included in the study were spinal arthrodesis, anterior or posterior interbody techniques, and posterior or posterior lateral fusion techniques. The location of the surgery (cervical, thoracic, or lumbar) and the number of levels fused were indicated as a covariate. Neurosurgeons performed relatively more on the cervical spine (32% vs. 24%) compared to orthopedic surgeons who performed lumbar spine operations more frequently (76% vs. 65%). There was no statistically significant difference in the number of levels fused or the operational approach utilized. There was no clinically significant distinction in the majority of perioperative outcomes between orthopedic surgeons and neurosurgeons [57].

Chun et al. analyzed the correlation between spine surgeon demographics and complication rates after elective spinal arthrodesis. They created a database using publicly accessible data from the Centers for Medicare and Medicaid Service, covering various procedures like pre-sacral interbody, posterolateral, posterior, and combined posterolateral and posterior techniques. Complication rates were determined using the ProPublica Surgeon Scorecard, focusing on mortality during the original hospital stay or readmission within 30 days for poor surgical outcomes. The study included 2,110 spine surgeons who performed 142,863 elective spine fusion procedures on 125,787 Medicare patients between 2011 and 2013. The adjusted complication rates showed no statistically significant difference across surgical specialties. However, the authors reflected upon some limitations of the study. The study collected data only from publicly available data. All procedures were combined into a single category according to the International Classification of Diseases (ICD) coding, whereas there could be different adaptations made in performing different procedures. Different surgical techniques may lead to different complication rates, which could not be accurately studied. This study only included surgeons who treat Medicare patients and not all spine surgeons [58].

Methodological limitations include the studies’ retrospective nature. In addition, the NSQIP database’s role is to capture data about various surgical subspecialties. Despite minimizing biases and confounding of different variables, there are potential concerns regarding selection bias and reliability of data collection. Per ACS NSQIP, audit reports have revealed an overall disagreement rate of <2%. Thus, data points are not specific to spine surgery. The variables of interest specific to NS, including postoperative symptom relief, and rates of neurological complications, such as postoperative sensory loss, weakness, or cerebrospinal fluid leak are not included in the database. Relevant data points such as specific radiographic findings or quality-of-life measures are not collected in the database. The NSQIP database also includes outcomes in the 30-day postoperative period only, limiting the ability to assess midterm and long-term outcomes. Furthermore, potential variables such as hospital volume, geographic location, quality-of-life measures, and skill level of individual surgeons were not mentioned in the NSQIP. The potential for residual confounding cannot be eliminated due to the statistical models being limited to the variables recorded in the NSQIP. Only a fraction of hospitals participate in the ACS NSQIP, rendering it incomplete in certain contexts [45,46,52].

Percutaneous kyphoplasty

A study analyzed the relationship between the specialty of a spine surgeon and short-term adverse events in patients who underwent PKP. Among the patients, 10.2% recorded one or more postoperative complications and 9.5% had unplanned readmissions within the 30 postoperative days. Unplanned reoperation rates and readmission rates between the two specialties were similar. OS patients required more frequent blood transfusions postoperatively within 72 hours compared to NS patients. African Americans needed more transfusions compared to Caucasians in the OC. However, patient with preoperative pulmonary disorder, or disseminated cancer has higher transfusion rates within the NC. Higher readmission rates were attributed to age 85 and above in the NC, and urinary system disorders in the OC. Patients who underwent thoracic PKP needed fewer blood transfusions but higher readmission rates compared to lumbar PKP patients [59].

Adult spinal deformity (ASD)

A study by McDonald et al. analyzed ASD treatment procedures by neurological and orthopedic surgeons using the Pearl Driver Mariner database between 2010 and 2019. The study assessed baseline characteristics, surgical methods, and volume, as well as cost, reoperation rate, and complications. Most deformity surgeries were performed by orthopedic surgeons. Orthopedic procedures had lower costs than neurologic procedures. While orthopedic surgeons perform most of the adult deformity procedures, neurological surgeons are increasingly operating on elderly patients with more comorbid conditions. Neurosurgeons also performed guided or robotic surgeries, three-column osteotomies, and multilevel arthrodesis more frequently. This study faced limitations due to intrinsic limitations in administrative databases, including the absence of necessary outcomes and conditions in medical records, and the lack of generalizability. The costing information was incomplete and misrepresented posing a pertinent challenge [60].

Spinal metastases

Malik et al. conducted a study that included surgeries performed by orthopedic and neurologic surgeons for patients undergoing fusions, laminectomies, or osteotomy/corpectomy for spinal metastases. Variables collected include baseline clinical characteristics, such as age, gender, race, region, plan, Elixhauser comorbidity index (ECI), primary cancer, and type of surgery. Complications included sepsis, pneumonia, UTI, acute renal failure, emergency room visits, implant problems, revision surgery, 90-day readmissions, and 90-day mortality. No statistically significant differences were noted between the two specialties in terms of complications. A surgeon's specialty does not influence intermediate-term complications following surgical intervention for spinal metastases. Limitations include those introduced by coding or billing errors, commercial insurance claims being unrepresentative of the general population, and lack of information about physician fellowships, primary practice, and the total number of years in practice. Lack of identification of approach (anterior vs. posterior vs. circumferential) and number of levels fused posed certain limitations. The authors proposed that future studies need to include more comprehensive data including the degree of tumor invasiveness, spinal parameters, and degree of deformity among other aspects mentioned previously. The use of administrative databases renders the acquisition of comprehensive information difficult, including important data such as adjunct therapies for cancer treatments [61].

Isolated spinal fractures

A retrospective study by Myers et al. conducted through trauma audit and research network (TRAN) compared the management of isolated spinal fractures between OS vs. NS at a single center in the United Kingdom. Out of 465 patients managed in total, 266 procedures were performed by neurosurgeons and 199 by orthopedic surgeons, which included 615 fractures. Twenty-seven patients from the NC suffered spinal cord injury, while 14 patients suffered spinal cord injury among the OC group. ICU stay and length of hospital stay were comparable between the two groups. Thirteen patients managed by NS contracted postoperative UTIs compared to nine patients managed by OS. However, 13 and 18 patients suffered the complication of lower respiratory infections among NC and OC respectively. Spinal complications like vertebral collapse, foot drop, and pseudoarthrosis were four in NC and 10 in OC. Overall complications rate was higher in the orthopedic cohort compared to the NC. Nine patients from NC and eight from OC resulted in mortality. They concluded that the difference between OS versus NS in the management of spine fractures exists, and this needs to be studied in detail owing to the differences in training and skills to standardize the management protocols [62].

Discussion

Specific types of surgeries are occasionally not integrated between both specialties and sometimes fall under the purview of either OS or NS. Traditionally, OS training emphasized bone biology and healing concerning principles of mechanical alignment, stability, and deformity along with spinal instrumentation. Orthopedic surgeon that has a specialized focus on primary spine surgeries target addressing the disarrangement of the spine's structure and stability. This includes conditions such as scoliosis, herniated discs, and spinal stenosis. A neurosurgeon specializing in spine surgery will treat degenerative conditions and disorders that include the nervous system within and around the spine such as spinal cord compressions and tumors. Cases involving more focus on cerebral structures and intradural surgeries were the emphasis of NS training. These subtleties are more likely to be learned by trainees with fellowship cross-training. Spine pathologies are a spectrum where both these principles must be applied together for optimal outcomes of the diseases during treatment. Collaboration in training routes between orthopedic and neurological surgeons has increased in recent times, particularly during fellowship training. This partnership is believed to have resulted in both orthopedic and neurological surgeons being more skilled at treating spinal deformities. Because neurosurgeons and orthopedic spine surgeons have traditionally had diverse training backgrounds, spine patients are likely to benefit from the distinct skill sets and perspectives that each discipline offers. The institutionalization of the collaboration between neurosurgeons and orthopedic spine surgeons may enhance spine patient outcomes. It is essential to note the emphasized discussion on the merits and demerits of the comparative effectiveness of each specialty as it translates to results between different subspecialties. However, improvements in outcomes are more probable in high-volume clinics when neurosurgeons and orthopedic surgeons collaborate [63,64].

Some noteworthy differences were found among patient demographics treated by two specialties. It is observed that the patients operated on by NS tend to be older and functionally dependent compared to patients operated on by OS [45,52,60]. However, in a study conducted on isolated spinal fracture management, it was found that patients operated on by NS were younger and functionally independent [58]. Although it may be clinically insignificant, some studies found that a lower proportion of Caucasians constituted the patient cohort treated by OS [49,58]. However, one study revealed that the patients operated by OS were found to belong to white ethnicity and constituted of higher proportion of males [61]. It was a consistent finding that patients being operated on by NS had many comorbidities [45,52,53,58,60]. The mean American Society of Anesthesiologists (ASA) class was found to be higher in patients who were being operated on by NS [45,50,53,54].

One study showed that NS performed more procedures on the cervical spine, whereas OS performed more procedures on the lumbar spine [50]. In another study on surgeon specialty differences among elective spinal procedures, it was revealed that neurosurgeons extensively operated on patients with spondylosis and disk displacement whereas orthopedic surgeons operated frequently on patients with disk degeneration [57]. It was found that patients managed by OS were more likely to undergo operative procedures than the patients managed by NS. NS was observed to apply a conservative approach frequently when compared to OS. It was also observed that in the management of patients with thoracolumbar fractures, the percutaneous approach was significantly more employed by neurosurgeons. Although the NS admitted more cases, it is observed that patients getting admitted under OS had higher chances of undergoing a surgical procedure [12]. These differences can be attributed to the differences in specialty training where orthopedic surgeons tend to focus on surgical correction and neurosurgeons tend to prefer a relatively minimally invasive approach.

It was found that procedures conducted by OS had significantly lower average costs for the procedure [56,60]. Perception of surgeon specialty and differences in instrumentation could be the factors contributing. On the other hand, one study found that 90-day costs were similar between NS and OS, but it was found that the surgeon reimbursement was lower for OS compared to NS [47]. A consistent finding among multiple studies was that NS had a longer operative time [46,50,52,53]. This longer operation can be attributed to the fact that neurosurgeons operated on patients with more comorbidities and higher ASA scores, which can complicate the procedure.

A significantly consistent finding among the studies included was the higher blood transfusion rates of patients operated on by orthopedic surgeons [46,48,50,52,54,57,58]. In one study, it was observed that patients who underwent an operation by OS had twice the odds of receiving perioperative transfusion compared to patients who underwent an operation by NS [57]. It was observed that patients who were operated on by neurosurgeons had shorter hospital stays [46,52,57]. One study depicted that overall in-hospital complications were more prone to occur in patients operated on by orthopedic surgeons. The complications were found to have a skewed distribution, a few complications such as sepsis are more common in patients operated by orthopedic surgeons, while complications like deep vein thrombosis and UTIs were found to be more common among patients operated on by neurosurgeons [45,52,54]. In a study, it was found that orthopedic surgeons were found to have lower odds of wound complications, whereas neurosurgeons were found to have lower odds of dural tears. The author attributed this finding to differences in specialty training where orthopedic surgeons were trained to have a strong emphasis on bone and soft tissue healing, while neurosurgeons were more focused on intradural processes and cerebrospinal fluid management [47]. Other than the aforementioned complications, most studies found no statistically significant difference in outcomes of spinal procedures by neurosurgeons and orthopedic surgeons [45,46,47,48,49,51,53,55,61].

Generally, the re-admission and re-operation trends among the studies included showed lower re-admissions and lower re-operation rates for cervical procedures by neurosurgeons and higher re-admission and re-operation rates for lumbar procedures by neurosurgeons [45,52,56].

A few studies showed small variations in terms of blood loss and operative timings. Because neurosurgeons spend a substantial amount of their training and surgical practice doing intracranial procedures, where perfect hemostasis is crucial to avoid devastating results, including death, they may spend more time controlling blood loss during spine surgeries. This might result in longer operational times and reduced transfusion rates [65]. Many studies showed a cutdown in operative time blood loss intraoperative complications and hospital stays when surgeons of both specialties collaborate and attend the surgery [24]. There was no statistical difference in wound infection rates between spine surgeries performed by two specialties [66].

The significance of neurosurgeons and orthopedic surgeons sharing comparable evidence-based principles in spine pathology decision-making and surgical technique cannot be undermined. Improved teamwork and precise coordination can lead to demonstrated gains in patient care in spinal pathologies [67]. Similar approaches to spinal parameters and surgical methods, as well as techniques for complex spinal surgeries, must be maintained. This kind of surgical harmonization will aid in achieving superior results.

One of the challenges faced includes time constraints due to the busy schedules of two spine surgeons, making it difficult to coordinate combined outpatient clinics and surgeries. Another obstacle is the difficulty in identifying the second surgeon's reimbursement, impeding the widespread use of dual-attending techniques at most deformity clinics [68]. Regarding financial implications, a collaboration of two surgeons is expected to increase expenditures in the short term [69,70]. However, as the number of major complications and resultant hospital stays is expected to decrease, the cost-benefit is anticipated to be realized in the long term [71,72]. We foresee a need for cost-effective assessments to determine the benefit of collaboration between orthopedic surgeons and neurosurgeons in complex spinal procedures.

A Seattle team-based concerted collaborative approach in managing complex spinal surgeries is a relevant example reflecting improved results. They showed that a multidisciplinary team with a dual-attending surgeon, one neurosurgeon, and one orthopedic surgeon (both being primary surgeons), a live preoperative screening conference, and an intraoperative protocol to manage coagulopathy will significantly decrease the perioperative complications and improve outcomes of spinal reconstructions for adult spinal deformities. The majority of these patients with complex spinal deformity due to kyphosis, scoliosis, or degenerative deformity with the instability of the lumbar spine had elevated operative risks, including pulmonary restriction, age-related risks, deep vein thrombosis, coagulopathy, cardiovascular complications, and prolonged surgery due to complexity of the procedures. Integration of multidisciplinary protocol and two-surgeon approach showed decreased complications in terms of mortality and morbidity and enhanced patient safety [73].

True competence is increasingly recognized to require appropriate assessment and reaction to one's technical and cognitive constraints [74]. Dual attending provides for close professional interaction, open case-based discussions, and mutual learning across disciplines. When both surgeons are at the same skill level, this can be a win-win situation; however, it may even be better when each team member has certain strengths from which the other surgeon learns.

When strategies are agreed upon, such as positioning both surgeons diagonally, initiating the dissection, and determining the cephalad or caudal instrumentation placement, an increase in coverage of the operative field can be optimized. It has been observed that a second attending surgeon can better predict the following technical stage for difficult operations. Both surgeons must be familiar with navigation or freehand screw placement procedures, as simultaneous insertion would be problematic if both relied on C-arm fluoroscopy or the same navigation gear [75]. Aside from the quantitative advantages that a dual-attending method may provide, nontechnical components of surgery that we discovered to be favorably influenced include teamwork, shared decision-making, and communication [76]. Another benefit cited in certain studies was a reduction in effort and stress for each surgeon [68]. In long and difficult operations that require a significant amount of attention, the dual-attending technique allows for alternating brief pauses, which are important to minimize exhaustion and maintain good performance, mirroring the pilot and co-pilot scenario in long-haul flights [72].

We foresee a dire need for studies involving an integrated approach of orthopedic surgeons and neurosurgeons or multidisciplinary involvement in terms of improvements in diagnostic accuracies, treatment delay, reducing perioperative morbidity, and overall improved outcomes and cost efficiency in the long term about spinal disorders. Incorporation of experienced orthopedic and neurosurgical guidance in training for spine fellowship may be considered to expand the individual expertise and skills from each specialty. This is more so in academic institutions and high-volume centers where spine fellowship training is provided, which may improve safety, quality, and outcomes. Stratifying the real need for an integrated approach for procedures, traditional mindset, financial implications, and optimal resource utilization to counter the challenging questions and overcome the obstacles in this pathway.

## Conclusions

Spine surgery and its related field have seen many technical and surgical advancements over the years owing to the contributions by both orthopedic surgeons and neurosurgeons. There are indeed fundamental differences in residency training and practice between the two specialties. However, the overwhelming lack of statistically significant difference in treating spinal disorders in terms of intraoperative complications is a testament to the ability of surgeons of both specialties to conduct a plethora of procedures. Therefore, it can be stipulated that patients undergoing conventional procedures can confidently select a surgeon from either surgical branch.

While neurosurgeons are trained to avoid blood loss for complicated spine procedures, eventually increasing the operative time, orthopedic surgeons can bring their expertise in reducing operative time. Nonetheless, collaboration, despite time restrictions and financial implications, is beneficial for better patient outcomes and is the way forward for complex spinal procedures. Having the presence of both types of surgeons allows for professional interaction, mutual learning, and cross-pollination of ideas, which could eventually lead to a mutualistic benefit. With the advent of super subspecialties, it is imperative to train surgeons incorporating diverse expertise from various areas of surgery and devise standard practice guidelines to alleviate complications.
